# Phase-specific expression of an insulin-like androgenic gland factor in a marine shrimp *Lysmata wurdemanni*: Implication for maintaining protandric simultaneous hermaphroditism

**DOI:** 10.1371/journal.pone.0172782

**Published:** 2017-02-24

**Authors:** Dong Zhang, Min Sun, Xin Liu

**Affiliations:** 1 Key Laboratory of East China Sea & Oceanic Fishery Resources Exploitation and Utilization, Ministry of Agriculture, Shanghai, P. R. China; 2 East China Sea Fisheries Research Institute, Chinese Academy of Fishery Sciences, Shanghai, P. R. China; Evergreen State College, UNITED STATES

## Abstract

**Background:**

Shrimp in the genus *Lysmata* have a unique and rare sexual system referred to as protandric simultaneous hermaphroditism, whereby individuals mature first as male (male phase), and then the female function may also develop as the shrimp grow, so that the gonad is able to produce both eggs and sperms simultaneously, a condition called simultaneous hermaphroditism (euhermaphrodite phase). To date, the mechanisms of sex control in this sexual system still remain poorly understood. Many studies indicate that an insulin-like androgenic gland factor (IAG) is involved in controlling sex differentiation in gonochoric crustaceans, but its role in the protandric simultaneous hermaphrodite is still not clear.

**Results:**

To determine whether an IAG is involved in sex control in the hermaphrodite, here we, for the first time, cloned the *IAG* gene cDNA sequence from *Lysmata wurdemanni* (termed *Lw-*IAG: *L*. *wurdemanni* insulin-like AG factor), a protandric simultaneous hermaphroditic shrimp. The IAG contains an open reading frame of 528 bp, corresponding to 176 amino acids, which consists of a signal peptide, B chain, C peptide, and A chain. The organization is similar to the *IAG*s found in other decapods. The *IAG* gene was expressed in both male and euhermaphrodite phases, but the expression level was significantly higher in the male phase than in the euhermaphrodite phase. Immunofluorescence analysis and Western Blotting revealed that the IAG protein was expressed in the androgenic gland, and its expression level was higher in the male phase than in the euhermaphrodite phase.

**Conclusions:**

Data presented here suggest that the *IAG* gene may be a factor controlling sex in the protandric simultaneous hermaphrodite, and that the euhermaphrodite phase is maintained by reduced gene expression, i.e., the presence of the androgenic gland (or the androgenic hormone it produces) completely inhibits ovarian development in the male phase, and incomplete degeneration of the androgenic gland in the euhermaphrodite phase results in simultaneous hermaphroditism. The findings presented in the current study can help to reveal how protandric simultaneous hermaphroditism evolved in crustaceans.

## Background

Protandric simultaneous hermaphroditism (PSH) is a rare sexual system that exists in some animals, such as flatworms [1], polychaetes [2], snails [3], and shrimp species in the genera *Lysmata* [4–6], *Exhippolysmata* and *Lysmatella* [7–8]. In shrimp with PSH, individuals mature first as male (male phase), and the female function may also develop as shrimp grow, so that the gonad is able to produce both eggs and sperms simultaneously, a condition called simultaneous hermaphroditism (euhermaphrodite phase) that has both male and female functions. The evolutionary significance of PSH and the mechanisms selecting for this sexual system are still two unresolved problems in evolutionary biology. Although the sex allocation theory has explained pure hermaphroditism (i.e. sequential hermaphroditism and simultaneous hermaphroditism) very well [9–12], there are still no proper models to explain intermediate or mixed sexual systems, like PSH, to the same degree [13,14]. Moreover, few empirical studies have dealt with the question of how PSH evolved in animals. Because the why and how questions are tightly associated, understanding the regulation mechanisms of sexual differentiation in animals with PSH is probably a good way to gain insight into the evolutionary significance of the sexual system. In the present study, we investigated the intrinsic factor controlling sex differentiation in a marine shrimp, *Lysmata wurdemanni*, which has a PSH sexual system.

In crustaceans, it is now well known that hormone(s) secreted by the androgenic gland (AG) determines the primary and secondary sexual characteristics in male individuals [15]. The absence of the AG results in exhibiting female secondary characteristics and vitellogenesis [16]; and the implantation of AG’s into immature females lead to sex reversal and the cessation of vitellogenesis [17–21]. Recently, genes expressed in the AG have been revealed using AG cDNA libraries prepared from some decapod crustaceans, such as crayfish, *Cherax quadricarinatus* [22], freshwater prawns, *Macrobrachium rosenbergii* [23], *M*. *lar* [24] and *M*. *nipponense* [25], and the Chinese shrimp, *Fenneropenaeus chinensis* [26]. Although the predicted structures of AG-specific insulin-like peptides in those crustaceans are similar and conserved, sequence similarity among the peptides is relatively low [27].

It has been suggested that part of a sexual shift in a protandric amphipod might result from insufficient AG hormone secretion [28]. A recent study on a sexual shift induced by the silencing of a single insulin-like gene in an intersex crayfish, *C*. *quadricarinatus*, further suggests that the AG-specific insulin-like factor (IAG) likely serves as a sex switcher that controls the maleness/femaleness balance in intersex organisms, even in sequential hermaphrodites [27]. Reasonably, we assume that PSH in crustaceans is probably regulated by the IAG as well.

To date, the mechanisms of sex control in the PSH sexual system remain poorly understood, although social-related sex change has been found in a few species, such as *L*. *wurdemanni*, a high-density-living species [29–32], and *L*. *secutillata* [28]. Hence, we hypothesize that the *IAG* gene plays an important role in the sex control of *Lysmata* shrimps, specifically, that the expression level of the *IAG* gene in the male phase is higher than that in the euhermaphrodite phase. As the first step to test the hypothesis, the cDNA sequence of the *IAG* gene was cloned from *L*. *wurdemanni* (*Lw-IAG*), and the *Lw-*IAG protein sub-cellular location was predicted. Moreover, the expression of the *Lw-IAG* gene and protein in the male and euhermaphrodite phases were compared in the present study.

## Materials and methods

### (a) Animals and tissue preparation

All the shrimp used in the present study were laboratory reared with protocols we developed [33]. Average total length (from the end of the telson to the tip of the rostrum) of male-phase individuals was 1.3±0.2 cm (n = 30), and euhermaphrodite-phase individuals averaged at 3.0±0.2 cm (n = 30). Shrimp were maintained in a recirculating system. The photoperiod was 14h light: 10h dark. The water temperature was maintained at 27°C, and water salinity was 30‰. They were fed frozen adult *Artemia* twice per day. Male-phase shrimp have well-developed appendices masculinae and appendix interna of pleopod 2 without setae. The euhermaphrodite-phase shrimp do not have appendices masculinae on the endopods of pleopod 2 [34]. Hence it is easy to identify the male- and euhermaphrodite-phase shrimp by observing the endopods of pleopod 2 [34]. Gonads with ejaculatory ducts and AGs of the male- and euhermaphrodite-phase shrimp were dissected, respectively, quenched directly into liquid nitrogen, and then stored at -80°C.

### (b) Coding sequence amplification of the *Lw-IAG* gene

Total RNA from the testicular part of the ovotestes was extracted with UNIzol reagent (Invitrogen) and treated with DNase I (Fermentas). cDNA was synthesized using l μg of total RNA and the First-Strand cDNA Synthesis Kit (Takara) according to the *KOD* DNA polymerase (Toyobo) instructions.

Degenerate primers were designed according to reported *IAG* gene sequences to amplify the gene domains, F1: 5'-CGTTGACTTYGACTGCGGYGACATA-3' and R1: 5'-CTCGATGCAATATTSGGCGACTTCC-3'. The amplification was performed with the Eppendorf profile: initial pre-denaturing at 94°C for 5 min, 30 cycles of denaturing at 94°C for 30 s, 65°C for 30 s and 72°C for 30 s. The amplified fragments were separated by electrophoresis on a 1% agarose gel, purified with DNA purification kit (Takara) and subsequently sequenced by Sangon Biotech Company (Shanghai, China).

The cDNA cloning was performed following the SMARTer^™^ RACE cDNA Amplification Kit User Manual using 1μl samples of cDNA. According to the conserved domains of the *Lw-IAG* gene, two primers, 3’RACE: F2 5'-GAAGTCTCCATCGCTCGTCCACCC-3' and 5' RACE: R2: 5'-AGTTTTCCTCACTCCGTCCCTCCG-3' were designed for the *Lw-IAG*. The 5' and 3' RACE cDNA were prepared with a SMARTer^™^ RACE cDNA Amplification Kit (Clontech). The primers mentioned above and the Universal Primers Mix provided in the kit were used to amplify the *IAG* gene. Amplification was performed with the Eppendoff profile: initial pre-denaturing at 94°C for 5 min, 30 cycles of denaturing at 94°C for 30 s, 69°C for 30 s and 72°C for 1 min. The PCR products were analyzed by electrophoresis on a 1% agarose gel and subsequently purified with a DNA purification kit and sequenced as above.

### (c) Analyses of the nucleotide and deduced amino acid sequences

The nucleotide sequences and deduced amino acid sequences of the *IAG* were analyzed using the BLAST algorithm (NCBI, http://www.ncbi.nlm.nih.gov/BLAST/). Signal peptides, propetide cleavage site, N-linked glycosylation site and phoshorylation sites were predicted by CBS prediction servers (http://www.cbs.dtu.dk/services). PSORT II Protein Sorting Prediction tool was used to predict the *Lw-*IAG protein sub-cellular localization (http://psort.ims.u-tokyo.ac.jp/form.html). Sequence alignment of the *Lw-IAG* was carried out with known sequences of other species obtained by MEGAG software. A phylogenetic tree was constructed with MegAlign software [25].

### (d) Phase-specific expression of the *Lw-IAG* gene

RT-PCR was performed with an ABI Prism 7000 Sequence Detection System. Total RNA from the testicular part of the ovotestes of the male and euhermaphrodite phases was extracted with UniZOL and treated with DNase I (Fermentas). We synthesized cDNA using l μg of total RNA and the First Strand cDNA Synthesis Kit (Takara) according to KOD DNA polymerase (Toyobo) instructions. Real-time transcript levels of the *Lw-IAG* in different phases were obtained using the following primers: F4: 5’-CCAACACCTTCGCCTCCGTCTG-3’ and R4: 5’-GCGTCATGTGCTGAATCTCCTCCT-3’ with the SYBR Premix Ex Taq^™^ II (Takara). Primer for β-actin was F5: 5’-AGACCACCTACAACTCCAT-3’, R5: 5’-CTGCTTGCTGATCCACAT-3’. Reactions were incubated at 95°C for 10 min, followed by 40 cycles of 95°C for 10 s, 60°C for 30 s. Individual samples were run in triplicate. At the end of the PCR cycles, melting curve analysis was performed to validate the specific generation of the expected PCR products. The expression levels of the *IAG* gene were normalized to beta-actin and calculated using the 2^-ΔΔ*CT*^ method. Student’s *t*-test was applied to compare the expression levels of the *Lw-IAG* in different phases.

### (e) Immunofluorescence staining and Western Blotting

Post vas deferens with AG were carefully removed from male- and euhermaphrodite-phase shrimp and then fixed in 4% paraformaldehyde solution. After dehydration through an ethanol series (30-50-70-80-90-100%; 7 minutes each), the AGs were transferred to xylene, embedded in paraffin, and then sectioned at 4–6 μm thickness.

For monoclonal antibody preparation, recombinant *Lw*-IAG peptide was obtained by chemical synthesis and coupled with BSA. Six peptides (antigenic determinants) of the IAG protein were selected and synthesized according to the results of sequence analysis. Disulphide bridge formation was not observed. The cleaned peptide emulsified with Freud’s complete adjuvant was injected into mice with three replicates. Each replicate had seven injections, each at a two-week interval. After immunization, monoclonal antibody against the *Lw*-IAG was prepared using hybridoma technique followed by identification of IgG isotype and its affinity. Paraffin sections were deparaffinized and rehydrated, then incubated in citrate buffer (0.5 M, pH 6, 10 min in microwave oven) for antigen retrieval. Slides were cooled to room temperature and rinsed with phosphate buffered saline (PBS) (0.01M, pH 7.4). Blocking (1% BSA, 0.3% Triton X-100 in PBS) lasted for 45 min at room temperature followed by incubation with primary antibody (1: 500) at 4°C over night. Slides were rinsed with PBS three times and incubated with secondary goat anti-mouse IgG H&L Alexa Fluor 594 (1:800) for 90 min at room temperature. After PBS washing, slides were mounted with DAPI and imaged using fluorescence microscopy.

Western Blotting was performed with tissue samples to detect the expression of the IAG protein in male- and euhermaphrodite-phase shrimp. The samples were dissolved in RIPA buffer and homogenized using an electric homogenizer. After centrifugation at 12,000 *g* for 5 minutes at 4°C, the protein concentration in the clear supernatant was determined using a BCA protein assay kit (Beyotime). Proteins were separated using SDS-PAGE (15%) and transferred onto PVDF membrane (200 mA, 50min). For each lane, 20 μl protein sample with 20 μl buffer solution was added. The membrane was blocked for 2 h at room temperature with PBS supplemented with 0.1% Tween-20 and 5% BSA, then incubated over night at 4°C with primary antibody (1:500). GADPH antibody (Santa Cruz Biotechnology, 1:2000) was used as an internal reference to certify integrity. After rinsing three times with PBS containing Tween-20, slices of the sample were incubated with HRP-conjugated goat anti-mouse secondary antibodies (Jackson ImmunoResearch Inc, 1:5000) at room temperature for 2 h. After rinsing, an ECL chemiluminescence detection kit for HRP (Biotech Well Company, Shanghai) was used to reveal positive bands visualized after 1 min exposure.

## Results

### Amino acid sequence analysis of the *Lw-*IAG

**Fig 1:**
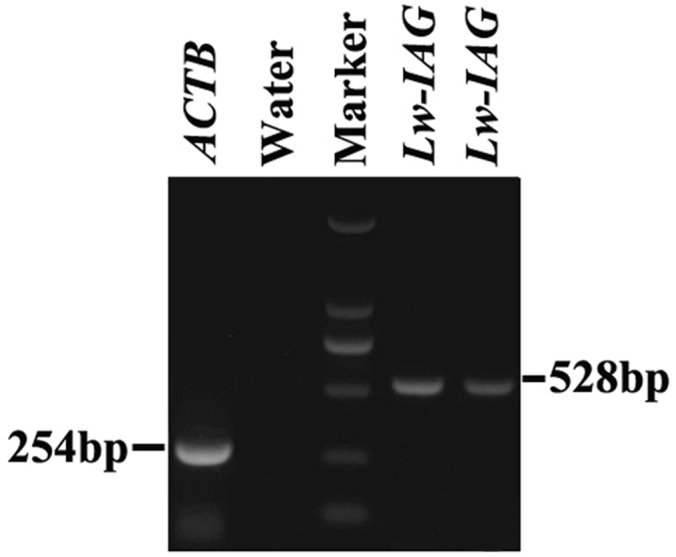
Amplified CDS of the *Lw-IAG* gene in *Lysmata wurdemanni*. RT-PCR shows the *Lw-IAG* gene’s CDS area. ACTB served as a loading control of total cDNA, and water without cDNA was employed as a negative control. A single band of 254bp was observed in ACTB sample. Bands at 528bp were observed in *Lw-IAG* samples but not in the water sample.

**Fig 2:**
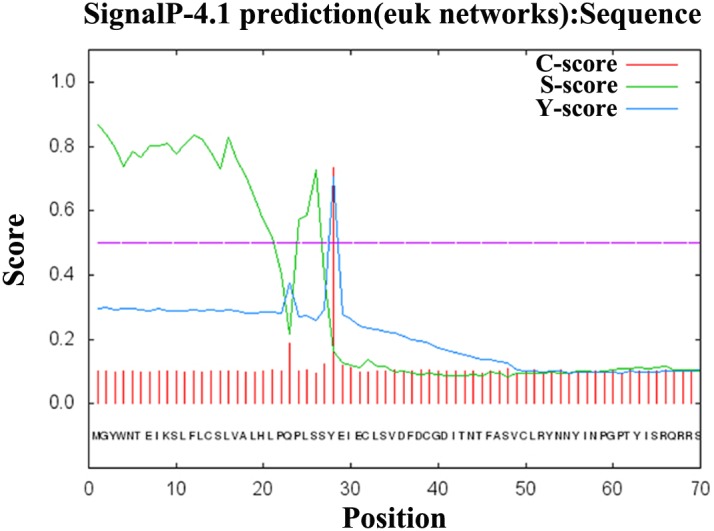
Prediction of the *Lw-*IAG signal peptide in *Lysmata wurdemanni*. The *Lw*-IAG protein belongs to hydrophobic protein, which consists of s a signal peptide, B chain, C peptide and A chain, and six conserved Cys residues. The *Lw*-IAG protein contains one O-glycosylation site, five phosphorylation sites and one N-glycosylation site. The cleavage site of the signal peptidase is between the 27^th^ and 28^th^ amino acids.

**Fig 3:**
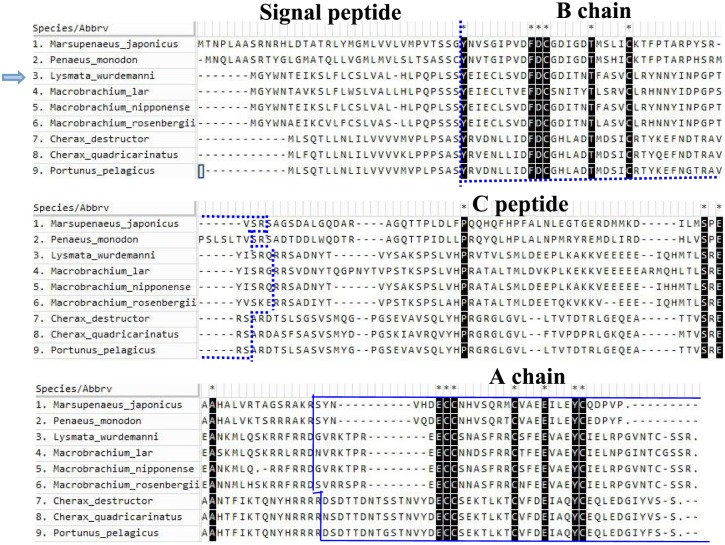
Multiple sequence alignment of deduced primary amino acid sequences of decapod *IAGs* using Clustal W. *Cherax quadricarinatus*: ABH07705.1; *Cherax destructor*: ACD91988.1; *Macrobrachium rosenbergii*: ACJ38227.1; *Portunus pelagicus*: HM459854.1; *Penaeus monodon*: ADA67878.1; *Marsupenaeus japonicus*: BAK20460.1 *Macrobrachium lar*: BAJ78349.1; *Macrobrachium nipponense*: AGB56976.1. The amino acid sequence of *Lw-LAG* is indicated by an arrow. The boundary between signal peptide and B chain is represented by a dotted vertical line. Conserved amino acids are indicated in different colors. B and A chains are marked with dotted and sold lines, respectively. The asterisk indicates the positions of conserved Cys residues in all species.

The *Lw-*IAG ORF was 528 nucleotides in length (Fig 1), which encoded a putative polypeptide of 176 amino acid residues. The molecular weight of the *Lw*-IAG protein was 20.19 kDa with a theoretical isoelectric point of 6.44. Sequence analysis indicates that the *Lw-*IAG protein is hydrophobic and contains a signal peptide, B chain, C peptide and A chain, and six conserved Cys residues (Fig 2). The cleavage site of the signal peptidase is between the 27^th^ and 28^th^ amino acids (Fig 3). The *Lw-*IAG protein contains one O-glycosylation site, five phosphorylation sites and one N-glycosylation site. Sub-localization analysis predicts that *Lw-*IAG is located in both the cell nucleus and cytoplasm.

### Phylogenetic analysis of the *Lw-IAG* gene

**Fig 4:**
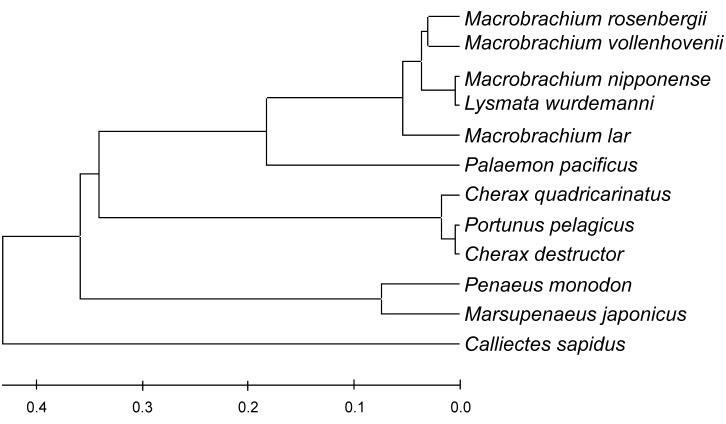
Phylogenetic tree built with the *IAGs* of eleven decapod crustaceans. The *IAG* protein sequences of crustaceans ranging from 170 to 185 aa were selected to establish a phylogenetic relationship. The amino acid sequence of the *Lw*-*IAG* has higher sequence similarity to that of *M*. *nipponense* (98%), *M*. *vollenhovenii* (86%), *M*. *rosenbergii* (82%) and *M*. *lar* (74%), respectively, than that of *Palaemon pacificus* (48%), *Penaeus monodon* (29%), *Marsupenaeus japonicus* (29%), *C*. *quadricarinatus* (30%), *C*. *destructor* (29%), and *Portunus pelagicus* (28%), respectively. X-axis represents the difference among the amino acid sequences.

The *Lw*-*IAG* encodes a putative polypeptide of 176 amino acid residues, so IAG protein sequences of crustaceans ranging from 170 to 185 aa were selected to analyze phylogenetic relationships (Fig 4). BLASTn results show that the amino acid sequence of the *Lw-IAG* has sequence similarity with *M*. *nipponense* (98%), *M*. *vollenhovenii* (86%), *M*. *rosenbergii* (82%), and *M*. *lar* (74%), *Palaemon pacificus* (48%), *C*. *quadricarinatus* (30%), *Penaeus monodon* (29%), *Marsupenaeus japonicus* (29%), *C*. *destructor* (29%), and *Portunus pelagicus* (28%). Phylogenetic analysis indicates that *L*. *wurdemanni* and *M*. *nipponense* belong to the same family, and are close relatives of *M*. *vollenhovenii*, *M*. *rosenbergii*, and *M*. *lar* (Fig 4).

### Phase-specific expression of the *Lw-IAG* gene

**Fig 5:**
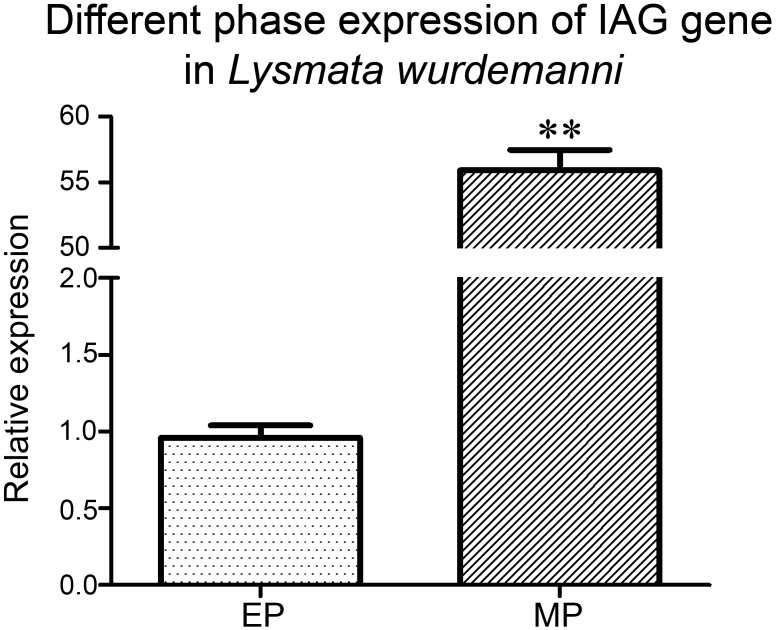
Expression of the *IAG* gene in *Lysmata wurdemann* in different phases. MP: male phase, E: euhermaphrodite phase. Relative expression levels of the *IAG* gene (2^-ΔΔ*CT*^) were quantified in male- and euhermaphrodite-phase shrimp by real-time PCR. Asterisks represent highly significant difference between two phases (Student’s *t*-test, n = 9, *P* < 0.01).

The *Lw-IAG* gene was detected in both male and euhermaphrodite phases by RT-PCR, however the expression level in the male phase was about 55 times higher than in the euhermaphrodite phase (Fig 5).

### Expression analysis of the *Lw*-IAG protein

**Fig 6:**
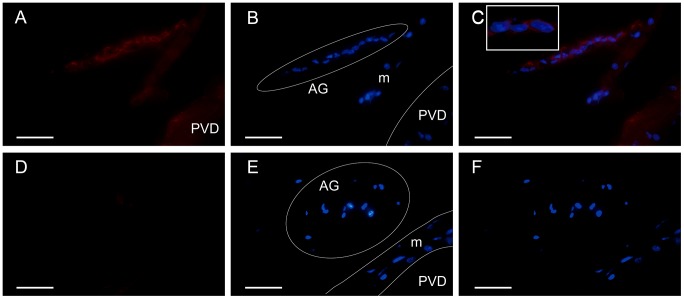
Immunofluorescence localization of *Lw*-IAG in *Lysmata wurdemanni*. Immunofluorescence was performed on sections of the androgenic gland from male-phase (A–C) and euhermaphrodite-phase shrimp (D–F). Specific signal (red) appears only in the AG cells of the male-phase shrimp. Nuclei (blue) were stained with DAPI, circles represent the area of AG cells. The white lines represent the boundary of posterior vas deferens (PVD). m: muscle layer of PVD. C: merged image of A and B; the enlarged view of AG cells is inset. The *Lw*-IAG was only expressed in cytoplasm based on the merged picture of the male-phase shrimp. F: merged image of D and E. Bar = 50 μm.

**Fig 7:**
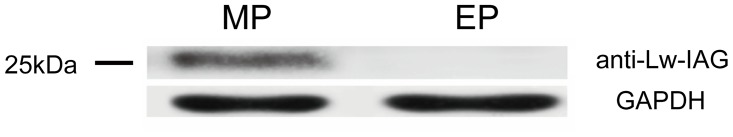
Western Blot analysis of the *Lw*-IAG protein in *Lysmata wurdemanni*. MP: male phase, EP: euhermaphrodite phase. A 25 kDa band only existed in the male-phase shrimp.

The IAG protein was located in the cell cytoplasm of the androgenic gland in male-phase shrimp. The positive signal (red) in the androgenic gland was too weak to detect in euhermaphrodite-phase shrimp (Fig 6). Western-Blot analysis found a 25 kDa band only in male-phase shrimp (Fig 7).

## Discussion

Insulin-like androgenic gland hormones have been demonstrated to control male sexual differentiation in crustaceans, mostly in dioecious and sequential hermaphroditic species [23, 27, 34]. In the present study, we isolated, for the first time, a gene encoding insulin-like androgenic gland hormone (*Lw-*IAG) from a protandric simultaneous hermaphroditic shrimp, *L*. *wurdemanni*. The *Lw-IAG* gene was expressed in both male and euhermaphrodite phases, but expression in the male phase was about 55 times higher than in the euhermaphrodite phase, suggesting that the *Lw-IAG* gene may play a role in controlling male sexual differentiation and maintaining PSH.

The *Lw-IAG* CDS region contains a signal peptide, B chain, C peptide, A chain, and six conserved cysteine residues, which is similar to the IAG of other crustaceans [5]. This indicates that the gene sequence of the IAG may be conservative in Crustacea.

Real-Time Quantitative PCR results showed that the *Lw-IAG* gene was expressed in both male and euhermaphrodite phases. However, the significantly higher expression level in the male phase suggests that the *Lw-IAG* gene may mainly work in the male phase, while reduced expression in the euhermaphrodite phase results in coexistence of both male and female functions. Morphological degeneration of the androgenic gland found in euhermaphrodite phase individuals of *L*. *wurdemanni* may be a reflection of functional repression, which is in accordance with the expression differences of the *Lw-IAG* gene in the different phases. Degeneration of the androgenic gland has also been found in a hermaphroditic shrimp, *Hippolysmata ensirostris* [7]. The disappearance of male external characteristics in the euhermaphrodite phase of *L*.*wurdemanni* during sex change indicates that the disappearance is synchronized with the degeneration of the androgenic gland [35], further supporting the idea that the *Lw-*IAG may play a role in regulating sex differentiation. Similar observations have been made in a sequential hermaphroditic shrimp *Pandlus borealis* [36] and isopod *Irona far* [37]. The difference between *L*. *wurdemanni* and the two protandric species, *P*. *borealis* and *I*. *far*, is that the androgenic glands of *P*. *borealis* and *I*. *far* totally degenerated in the late female phase [37]. Therefore, both morphological and molecular evidence suggests that PSH in *L*. *wurdemanni* is maintained through adjusting AG hormone secretion. The presence of the androgenic gland (or the androgenic hormone it produces) completely inhibits ovarian development in the male phase, and the incomplete degeneration of the androgenic gland in the euhermaphrodite phase results in simultaneous hermaphroditism. To completely understand how PSH evolved in crustaceans, the factors controlling AG degeneration should be investigated in the future.

Immunofluoresecence and Western Blotting demonstrated that the *Lw*-IAG protein was expressed in the cytoplasm, which is consistent with previous observations for *P*. *trituberculatus* and *M*. *rosenbergii* [23, 36]. However, it is inconsistent with the PSORT II Protein Sorting Prediction. It may be that dioecious and hermaphroditic crustaceans share the same regulatory mechanism of the IAG protein; and additionally, the nuclear localization sequence may become modified in the process of the IAG protein synthesis.

The IAG protein (Fig 7) was only detected in the male phase. The molecular weight was higher than the prediction in the present study, indicating that post-translational modification may exist during IAG protein synthesis. Molecular weight drift of the IAG protein has also been reported in *M*. *rosenbergii* [36]. According to the sequence analysis, the *Lw*-IAG protein may be glycosylated or phosphorylated, which might lead to the molecular weight drift; this assumption needs to be verified with more evidence in future studies. In the euhermaphrodite-phase shrimp, a very weak positive signal was detected during the immunofluoresecence experiment, and the expression level of the IAG protein was too low to detect with Western Blotting. The IAG protein is generally thought to be expressed only in male crustaceans [22, 23, 24, 25, 26, 27]. However, in *L*. *wurdemanni*, the euhermaphrodite-phase shrimp can act as an intermolt male [34], and although the *Lw*-*IAG* gene may be expressed in certain phases, the protein concentration in the euhermaphrodite phase may be too low to detect in Western Blot analysis.

## Conclusions

The *Lw-IAG* gene was expressed in both male and euhermaphrodite phases and the expression level in the male phase was about 55 times higher than in the euhermaphrodite phase, suggesting that the gene might function fully in the male phase, and the reduced expression in the euhermaphrodite phase might result in both male and female functions coexisting. These results, along with previous data from other decapod crustaceans, assures that *IAG* plays a role in controlling male differentiation in decapod crustaceans with dioecious, sequential hermaphroditic, and protandric simultaneous hermaphroditic sexual systems. The question then arises: “How does the *IAG* gene work in crustaceans with different sexual systems?” Apparently, the *IAG* gene is fully functional in the male of dioecious species. Complete repression of the *IAG* gene leads males to become female in sequential hermaphrodites; and and incomplete repression results in simultaneous hermaphrodites. In the future, causes of these differences in decapod crustaceans should be investigated.
